# Pediatric Mandibular Burkitt Lymphoma Initially Misdiagnosed as Odontogenic Infection: A Radiologic-Pathologic Correlation

**DOI:** 10.7759/cureus.106008

**Published:** 2026-03-27

**Authors:** Abhishek Singh, Avneesh Malviya, Sibgutulah Rashid, Mohit Singh, Manas Aeran

**Affiliations:** 1 Oral and Maxillofacial Surgery, Graphic Era Institute of Medical Sciences, Dehradun, IND; 2 Pathology, Graphic Era Institute of Medical Sciences, Dehradun, IND; 3 Oral and Maxillofacial Surgery, All India Institute of Medical Sciences, Patna, IND; 4 Dentistry, Graphic Era Institute of Medical Sciences, Dehradun, IND

**Keywords:** burkitt lymphoma, immunohistochemistry, mandibular swelling, pediatric lymphoma, r-copadm protocol

## Abstract

Burkitt lymphoma is a highly aggressive mature B-cell non-Hodgkin lymphoma characterized by MYC dysregulation and a high proliferative index. Although the sporadic form commonly presents with abdominal involvement, maxillofacial manifestations in children may mimic odontogenic infections, leading to delayed diagnosis.

We report a nine-year-old boy with a rapidly progressive unilateral mandibular swelling initially treated as a dental abscess without improvement. Imaging revealed an ill-defined osteolytic mandibular lesion with cortical erosion and soft tissue extension, raising suspicion for malignancy. Histopathology demonstrated monomorphic medium-sized atypical lymphoid cells with high mitotic activity. Immunohistochemistry confirmed B-cell lineage (CD20, CD10, BCL6, c-MYC) with a Ki-67 index of 95%, consistent with Burkitt lymphoma.

The patient was treated with rituximab-based multi-agent chemotherapy and showed a favorable response. This case highlights the importance of early radiologic-pathologic correlation and prompt biopsy in atypical pediatric mandibular swellings to enable timely diagnosis and management.

## Introduction

Burkitt lymphoma is an aggressive mature B-cell neoplasm defined by MYC gene dysregulation and an exceptionally high proliferative index. It is considered one of the fastest-growing human malignancies, with a tumor doubling time of approximately 24 hours, reflecting its highly aggressive clinical behavior. It represents a significant proportion of pediatric non-Hodgkin lymphomas and is notable for its rapid clinical progression. Based on epidemiologic and clinical features, three variants are recognized: endemic, sporadic, and immunodeficiency-associated [1,2].

In the sporadic form, abdominal involvement is most common; however, head and neck presentations, including maxillofacial and mandibular lesions, are well described in children. When arising in the jaws, the disease may present as a rapidly enlarging swelling with pain, tooth mobility, or soft tissue expansion, features that closely resemble odontogenic infection or inflammatory pathology [2,3]. Such overlap frequently leads to initial misdiagnosis and delay in definitive management.

Radiologic findings are often nonspecific and may demonstrate osteolytic bone destruction, cortical breach, and soft tissue extension, necessitating tissue diagnosis for confirmation [3]. Given the tumor’s potential for early dissemination, timely recognition and initiation of protocol-based chemotherapy are essential to achieve optimal outcomes [1]. This report highlights the diagnostic challenges associated with pediatric mandibular Burkitt lymphoma and underscores the importance of early radiologic-pathologic correlation in atypical jaw swellings.

## Case presentation

A nine-year-old boy was referred to our department with complaints of unilateral swelling in the right lower jaw associated with acute pain and occasional bleeding for two months. The patient had previously been treated by a general dental practitioner for a presumed dental abscess and space infection, without clinical resolution. Medical history was non-contributory.

Extraoral examination revealed facial asymmetry with a diffuse, bony hard swelling over the right mandibular body extending to the right submandibular region. The swelling was well defined, smooth-surfaced, and erythematous.

**Figure 1:**
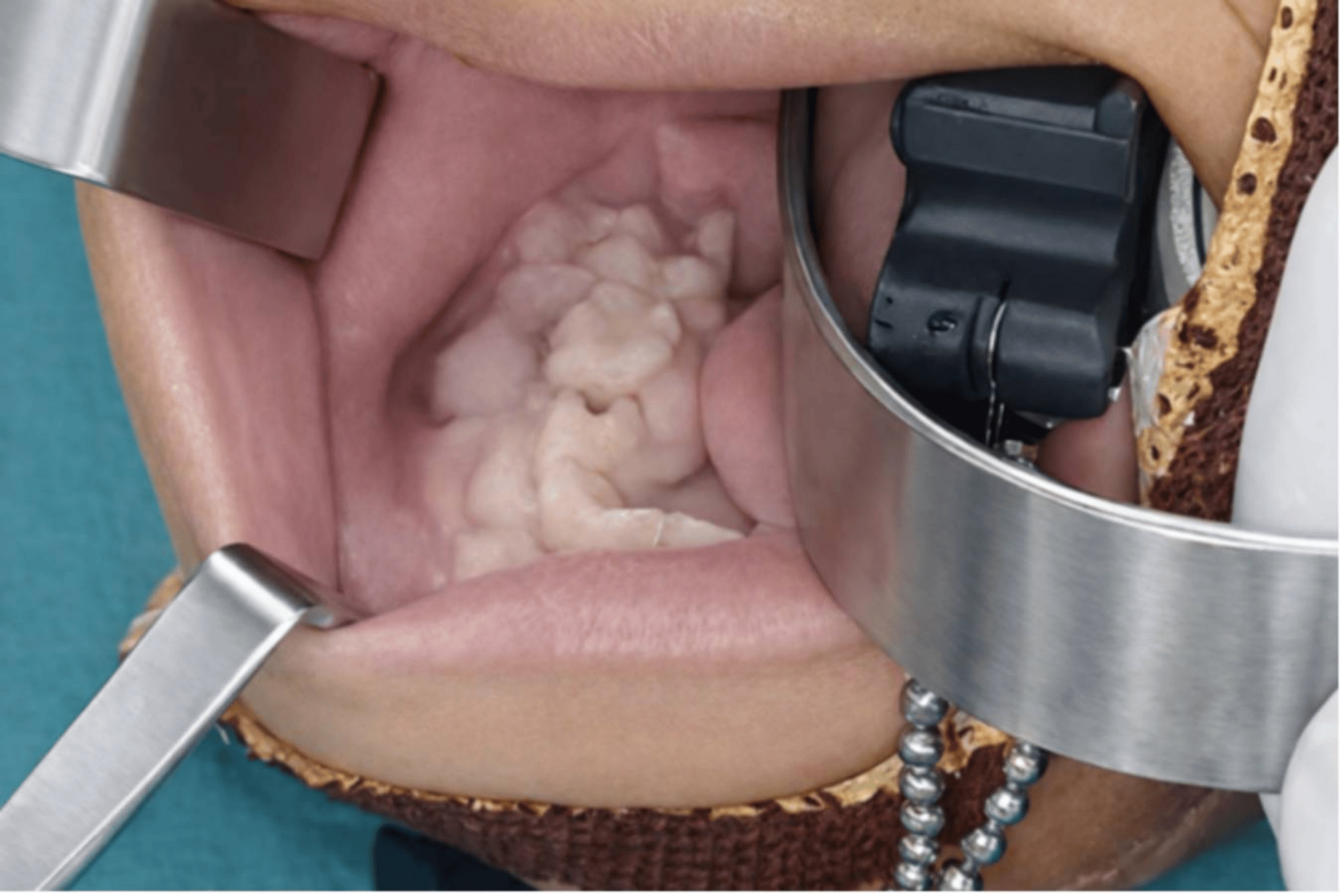
Intraoral examination showing a firm swelling in the right lower vestibule extending from 83 to 46.

Intraoral examination demonstrated a firm swelling involving the right lower vestibule extending from 83 (deciduous canine) to 46 (permanent first molar). The overlying mucosa appeared pinkish-white without ulceration (Figure 1). Teeth in the involved region exhibited mild mobility.

**Figure 2:**
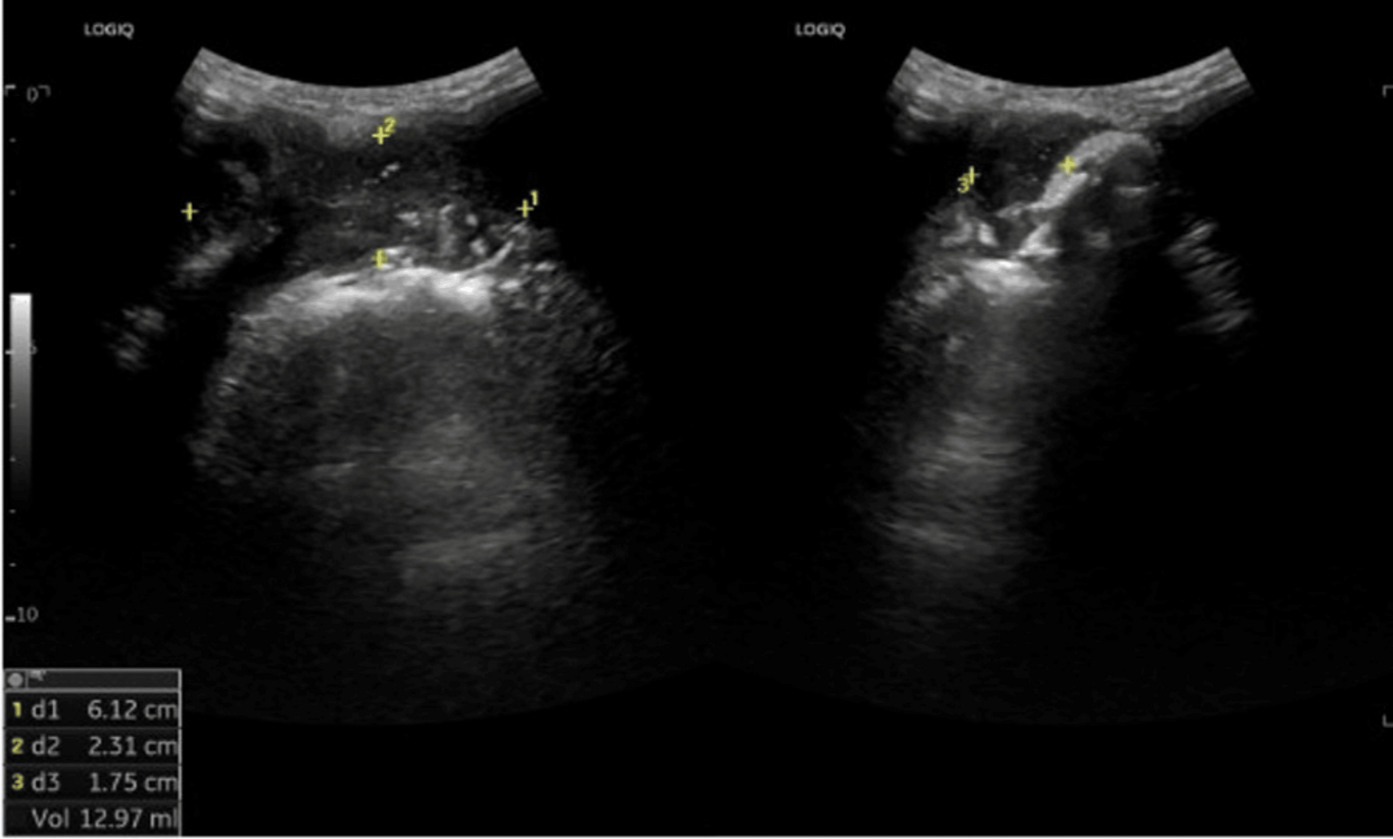
Ultrasound of the right submandibular region showing a heteroechoic collection (6.1 × 2.3 × 1.7 cm; ~13 mL) with internal echoes and posterior enhancement, suggestive of abscess.

USG of the right submandibular region revealed a well-defined hypoechoic to heterogeneously hypoechoic collection measuring 6.1 × 2.3 × 1.7 cm (~15 cc) in the right sublingual/submandibular space. A vascular hypoechoic subperiosteal component along the outer cortex of the right mandibular body was noted, continuous with the soft tissue lesion (Figure 2). An odontogenic abscess was considered; however, a neoplastic process could not be excluded.

**Figure 3:**
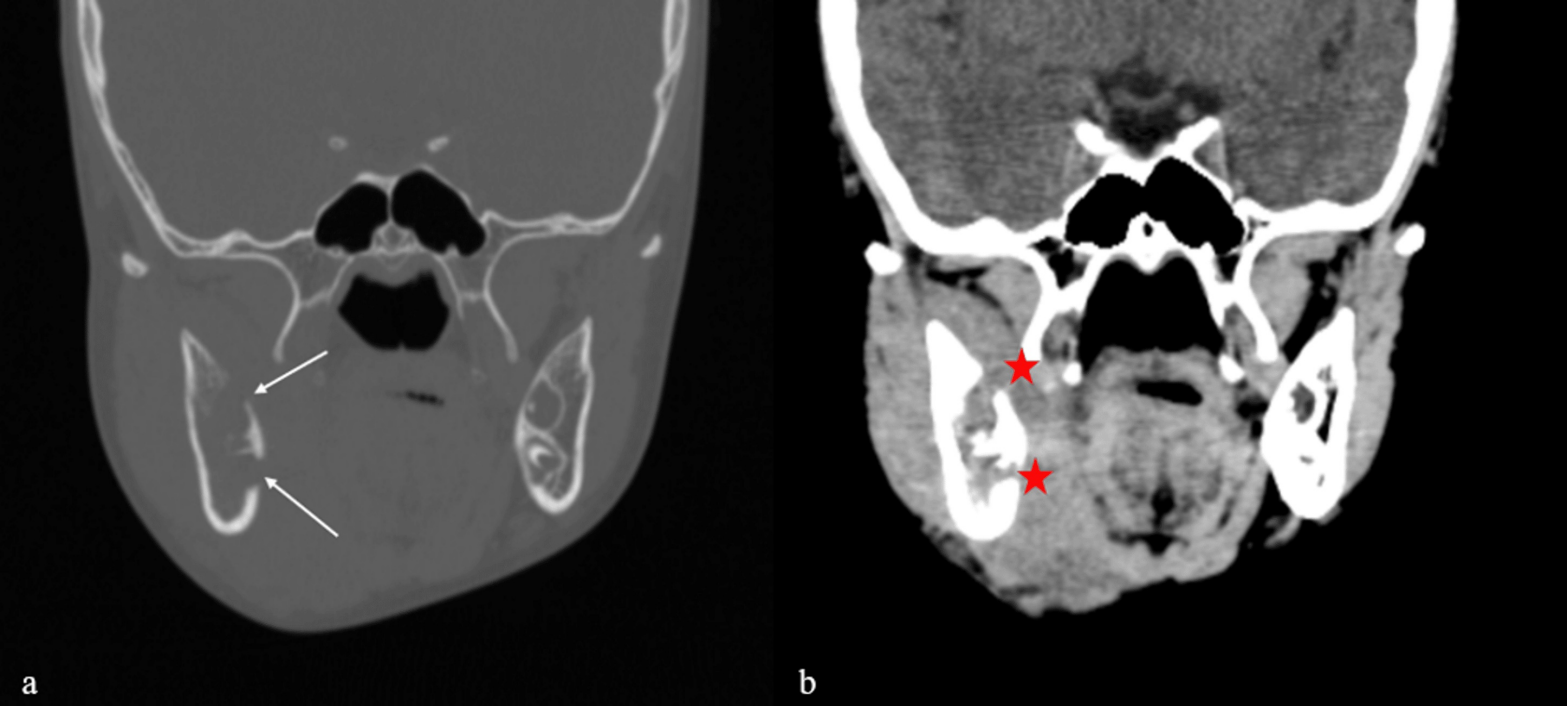
Non-contrast computed tomography (NCCT) of the face: (a) bone window demonstrating mandibular erosion with lingual cortical perforation (white arrows), (b) soft tissue window showing cortical breach with extension into the sublingual space (red stars).

Non-contrast computed tomography (NCCT) of the face demonstrated an ill-defined soft tissue lesion in the right submandibular region, with extension into the masseter and sublingual spaces. Bony erosion of the right mandibular body and ramus with mild periosteal reaction was present (Figure 3). Multiple level II lymph nodes were noted (largest 6.7 mm). Imaging favored a neoplastic etiology over chronic infection. Histopathological correlation was recommended.

**Figure 4:**
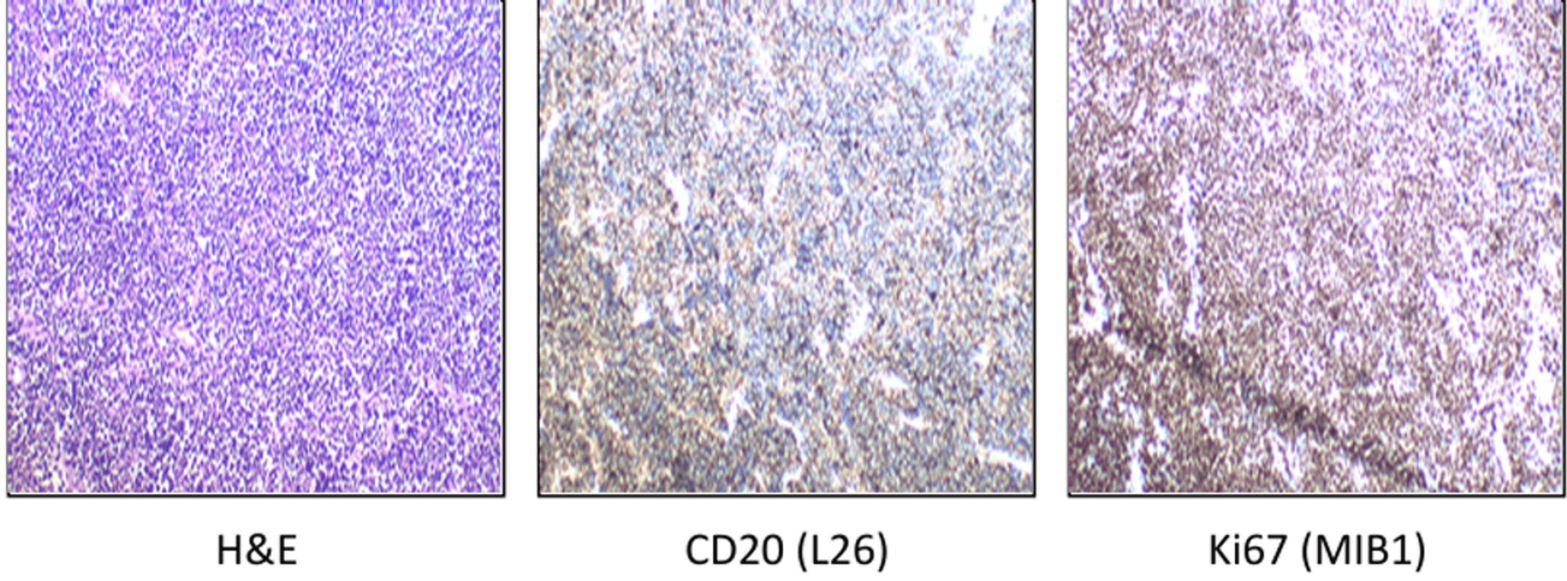
H&E section showing diffuse sheets of atypical lymphoid cells; immunohistochemistry reveals tumor cell positivity for CD20 (L26), with a high Ki-67 proliferative index.

**Figure 5:**
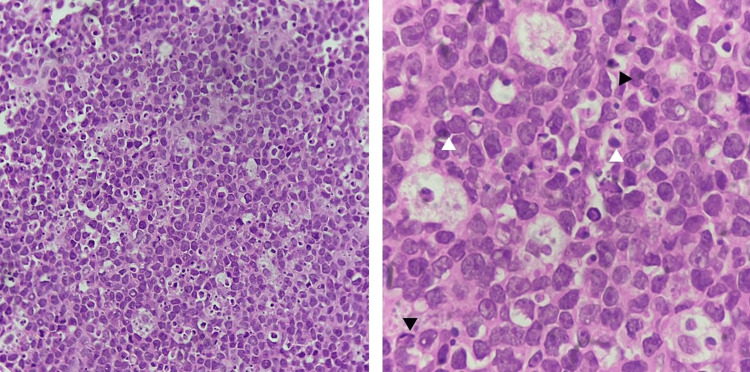
High-power photomicrograph (H&E, ×400) showing sheets of malignant B cells (white arrowhead) characterized by high a nuclear-cytoplasmic ratio, round-to-ovoid hyperchromatic nuclei, and frequent mitotic activity. A tingible body macrophage (black arrowhead) is also noted, contributing to the characteristic “starry-sky” appearance of Burkitt lymphoma.

Histopathological evaluation of both hard and soft tissue sections revealed an infiltrative malignant round cell neoplasm arranged in dyscohesive sheets and cords. The tumor cells were monomorphic, medium-sized, with a high nuclear-cytoplasmic ratio, round to ovoid nuclei, fine granular chromatin, inconspicuous to occasional nucleoli, and scant cytoplasm (Figures 4, 5). Brisk mitotic activity (29/10 HPF), numerous apoptotic bodies, karyorrhectic debris, and focal areas of necrosis were evident. Perivascular accentuation and infiltration between bony trabeculae were noted.

The histomorphological features were consistent with a malignant round cell tumor, with differential considerations including non-Hodgkin lymphoma, Ewing sarcoma, and small cell variant osteosarcoma.

Immunohistochemistry demonstrated tumor cell positivity for CD20, CD10 (weak), BCL6, MUM1, and c-MYC, and negativity for CD3, synaptophysin, desmin, MyoD1, NKX2.2, BCL2, TdT, SOX11, and CD138 [4,5] (Figures 4, 5). The Ki-67 proliferation index was approximately 95%, indicating a highly proliferative neoplasm [4,5].

Based on the integrated morphologic features and immunophenotypic profile, a diagnosis of high-grade B-cell non-Hodgkin lymphoma, consistent with Burkitt lymphoma, was rendered. Upon confirmation of this diagnosis, the patient was promptly referred to the Department of Pediatric Oncology for further staging workup and initiation of definitive therapy.

Systemic chemotherapy was initiated according to the R-COPADM protocol, consistent with contemporary pediatric mature B-cell lymphoma treatment regimens. The protocol comprised rituximab, cyclophosphamide, vincristine (Oncovin), prednisone, doxorubicin (Adriamycin), dexamethasone, and high-dose methotrexate. Rituximab was administered as per protocol schedule, and chemotherapy was delivered in inpatient cycles with standard supportive measures, including hydration, tumor lysis monitoring, hematological surveillance, and methotrexate level monitoring with leucovorin rescue as indicated.

The patient tolerated therapy without major treatment-limiting complications.

Response assessment was performed using fluorodeoxyglucose positron emission tomography-computed tomography (FDG PET-CT) after completion of scheduled cycles. Imaging demonstrated marked metabolic regression of the primary mandibular lesion and resolution of previously noted soft tissue involvement, consistent with a favorable therapeutic response. The patient remains under regular oncologic follow-up with ongoing clinical and radiological surveillance.

## Discussion

Burkitt lymphoma is a highly aggressive mature B-cell non-Hodgkin lymphoma characterized by deregulation of the MYC oncogene, most commonly through t(8;14)(q24;q32). It represents one of the most rapidly proliferating human malignancies, with a near 100% growth fraction reflected by a markedly elevated Ki-67 index. In the pediatric population, it accounts for approximately 30%-50% of non-Hodgkin lymphomas [1,2].

Although the sporadic variant most frequently involves the abdomen, maxillofacial involvement is well recognized in children. Mandibular presentations may clinically mimic odontogenic infection, periodontal abscess, or osteomyelitis, often leading to initial misdiagnosis and delay in definitive management [2,3]. In the present case, the patient was initially treated as a dental abscess, underscoring the diagnostic challenge posed by rapidly enlarging jaw swellings in children. Lack of response to conventional antimicrobial therapy should prompt reconsideration of the working diagnosis and early tissue sampling.

Radiologically, jaw involvement in Burkitt lymphoma typically manifests as ill-defined osteolytic lesions with cortical destruction and loss of lamina dura [6]. Cross-sectional imaging in this case demonstrated soft tissue extension with associated mandibular erosion and periosteal reaction, features that raised suspicion for a primary malignant bone tumor such as osteosarcoma or Ewing sarcoma. While imaging is critical for assessing the extent and local invasion, it remains nonspecific; definitive diagnosis relies on histopathological and immunophenotypic evaluation.

Histologically, Burkitt lymphoma is composed of monomorphic medium-sized lymphoid cells with a high nuclear-cytoplasmic ratio, brisk mitotic activity, and abundant apoptotic bodies, often producing a “starry-sky” appearance [5] (Figure 5). In the present case, the tumor demonstrated classic high-grade round cell morphology with extensive mitoses and necrosis. Immunohistochemistry confirmed B-cell lineage (CD20 positivity), germinal center phenotype (CD10, BCL6), c-MYC expression, and a high proliferative index (~95% Ki-67), while excluding small round blue cell tumors such as Ewing sarcoma (NKX2.2 negative) and rhabdomyosarcoma (Desmin, MyoD1 negative) [4]. The absence of BCL2 expression further supported the diagnosis [4-8].

Management of pediatric Burkitt lymphoma requires prompt initiation of intensive multi-agent chemotherapy. The addition of rituximab to established chemotherapy backbones, such as the R-COPADM regimen, has significantly improved event-free and overall survival rates [9,10]. With contemporary treatment protocols, survival exceeds 85%-90%, even in advanced-stage disease. Early diagnosis is therefore critical, as tumor doubling time is approximately 24 hours, and delays may result in rapid locoregional progression or systemic dissemination [11].

The favorable metabolic response observed on post-treatment FDG PET-CT in this case highlights the chemosensitive nature of the disease when treated appropriately. Long-term surveillance remains essential due to the risk of relapse, particularly within the first two years after therapy.

From a maxillofacial perspective, this case emphasizes that persistent, rapidly progressive mandibular swellings in children, especially those unresponsive to routine dental therapy, should prompt advanced imaging and early biopsy. Multidisciplinary collaboration between oral and maxillofacial surgeons, radiologists, pathologists, and pediatric oncologists is essential to ensure timely diagnosis and optimal outcomes.

Overall, this case reinforces the importance of maintaining a high index of suspicion for malignant pathology in atypical pediatric jaw lesions and demonstrates the critical role of integrated radiologic-pathologic correlation in achieving accurate diagnosis.

## Conclusions

This case highlights the importance of considering malignancy in rapidly progressive pediatric mandibular swellings, particularly when unresponsive to routine dental treatment. Early radiologic-pathologic correlation and prompt biopsy are essential for accurate diagnosis and timely initiation of appropriate therapy, which are critical for favorable outcomes in Burkitt lymphoma.
